# NMDA antagonists for treating the non-motor symptoms in Parkinson’s disease

**DOI:** 10.1038/s41398-018-0162-2

**Published:** 2018-06-15

**Authors:** Brigitte Vanle, William Olcott, Jaime Jimenez, Luma Bashmi, Itai Danovitch, Waguih William IsHak

**Affiliations:** 1 Department of Psychiatry and Behavioral Neurosciences, Cedars-Sinai Medical Center, Los Angeles, CA USA; 2 Medical College of Wisconsin, Wausau, WI USA; 3 David Geffen School of Medicine, University of California Los Angeles, Los Angeles, CA USA

## Abstract

Among patients with Parkinson’s disease (PD), depression is prevalent and disabling, impacting both health outcomes and quality of life. There is a critical need for alternative pharmacological methods to treat PD depression, as mainstream antidepressant drugs are largely ineffective in this population. Currently, there are no recommendations for the optimal treatment of PD neuropsychiatric symptoms. Given the dual antidepressant and anti-dyskinetic effects of ketamine and other N-methyl-D-aspartate (NMDA) antagonists for PD, this review aims to examine the current evidence of NMDA antagonists for treating neuropsychiatric symptoms, including memantine, amantadine, ketamine, dizoclopine, and d-cycloserine. A comprehensive literature search was conducted using the PubMed database. We also searched the following databases up to March 1, 2018: Ovid MEDLINE, PsycINFO, CINAHL, Google Scholar, Cochrane Central Register of Controlled Trials, and Cochrane Database of Systematic Reviews. The following keywords were used: NMDA antagonist and Parkinson’s disease. Two authors independently reviewed the articles identified from the search using specific selection criteria, focusing on studies of mood, psychiatric condition, depression, cognition, and quality of life, and the consensus was reached on the 20 studies included. There is a preliminary evidence that NMDA antagonists may modulate psychiatric symptoms in PD. However, current evidence of psychiatric symptom-modifying effects is inconclusive and requires that further trials be conducted in PD. The repurposing of old NMDA antagonists, such as ketamine for depression and newer therapies, such as rapastinel, suggests that there is an emerging place for modulating the glutamatergic system for treating non-motor symptoms in PD.

## Introduction

Parkinson’s disease (PD) is a chronic neurodegenerative disorder, characterized by motor and non-motor symptoms. The typical PD clinical manifestations are motor control impairments such as tremor, muscular rigidity, and bradykinesia^1^. However, there is a wide host of non-motor neuropsychiatric impairments implicated in PD, such as anxiety, apathy, cognitive dysfunction, and depression. These neuropsychiatric symptoms are especially debilitating and affect PD patients’ quality of life (QOL), yet may be under-reported^2^. For example, there is an evidence that depressive symptoms impair QOL and functioning more than any other PD motor and non-motor symptom^3^. Depressive symptoms are reported as high as 89% in the PD population^4^, with a mean reported prevalence rate of 40% in outpatient and 54% in inpatient settings^5^. Other non-motor symptoms affect QOL at the early stages of PD. In an exploratory drug trial, the most frequent psychiatric symptoms in PD patients were irritability (66.1%), depression (48.3%) followed by apathy (40.3%)^6^. While meta-analyses estimated more modest rates of 39% for depression (17% for major depressive disorder and 22% for minor depression)^5^, 31% for anxiety^7^, and 39.8% for apathy^8^. Symptoms of PD depression (PD-dep) are clinically different than symptoms in general depression, and more often portray severe irritability, sadness, dysphoria, pessimism, and suicide ideation^9^. The etiology of PD-dep is thought to be particularly influenced by interactions between exogenous (i.e., diagnosis of a chronic and disabling disease) and endogenous causes (i.e., loss of dopamine)^10^. The clinical manifestations of PD are elicited by the progressive loss of dopamine neurons. Disruption of dopamine^11,12^ and glutamate neurotransmitter systems is implicated in the heightened vulnerability and loss of dopamine neurons. The involvement of the glutamatergic system in modulating psychiatric disorders was first proposed by altered glutamate receptor expression^13^ and altered glutamate–glutamine levels in cerebrospinal fluid of patients with mood disorders^14^.

### Abnormal glutamate signaling

**Fig. 1:**
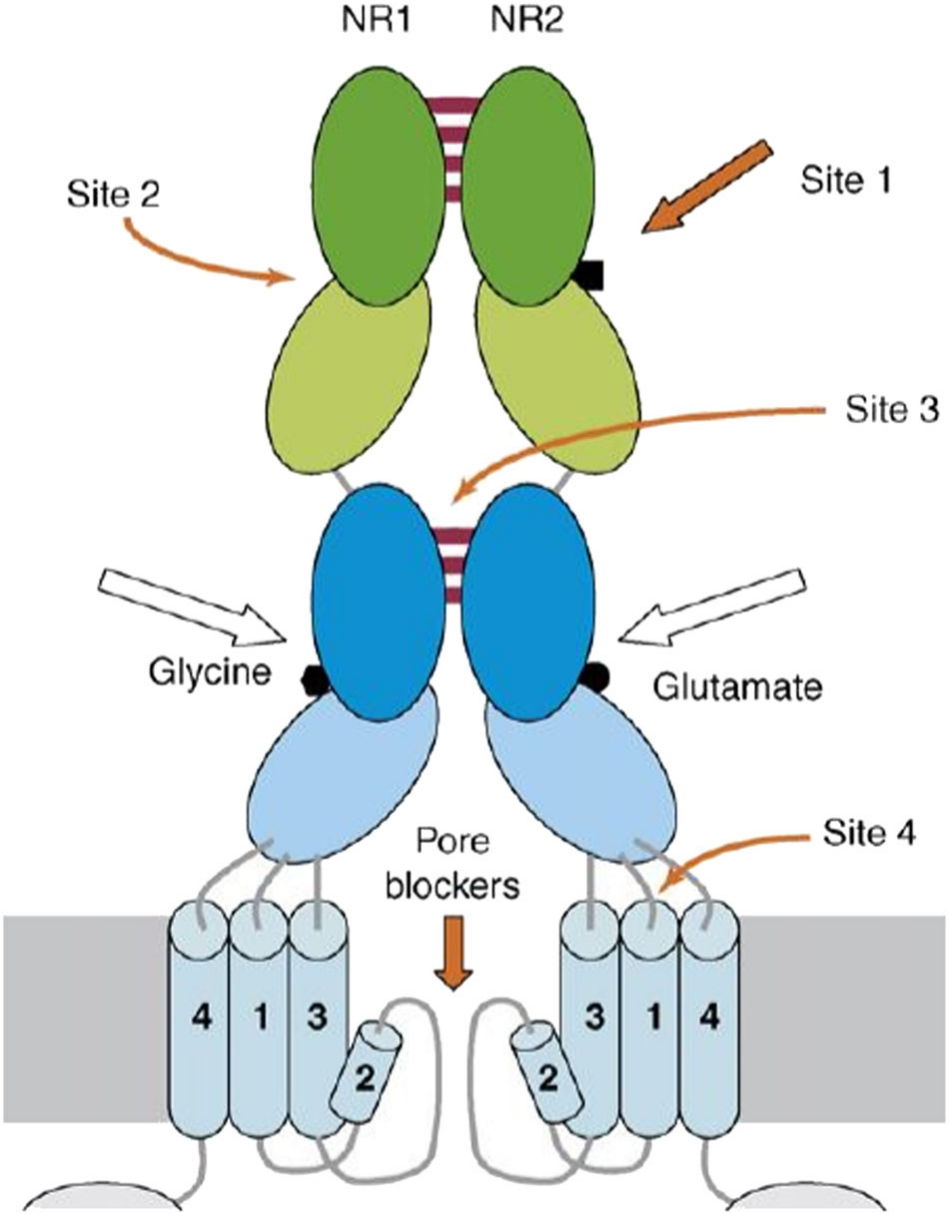
NMDA receptor consists of two heterodimers. Each heterodimer contains two extracellular subunits: NR1 and NR2. The NR1 subunit contains the glycine binding site, whereas the NR2 contains the glutamate binding site. Arrows show possible binding sites of uncompetitive/non-competitive antagonists (orange) and competitive antagonists (white)

Alterations in glutamatergic transmission are implicated in PD pathophysiology. The most characterized receptor in glutamate neurotransmission is the N-methyl-D-aspartate (NMDA) receptor. The NMDA receptor is composed of heteromeric subunits (NR1 and NR2), a glycine binding site, and a glutamate binding site^15^ (Fig. 1). The activation of NMDA receptors requires co-agonist binding of glycine/D-serine and glutamate; therefore, antagonists that disrupt co-agonist binding, effectively block the NMDA activity. The hyper-phosphorylation and resulting overactivation of NMDA receptors is well-established in PD; and is implicated in the worsening of dyskinesias^16–18^. The short-term L-DOPA-induced dyskinesias (LIDs) are a debilitating side effect of L-DOPA administration, and NMDA receptors are presumed to be partially responsible for LIDs^19^. The LIDs are a severe therapy-related complication in PD, and significantly impair QOL. Positron emission tomography (PET) images have confirmed an enhanced NMDA receptor activity in specific motor cortical areas of the brain during LIDs in PD patients^20^.

**Fig. 2:**
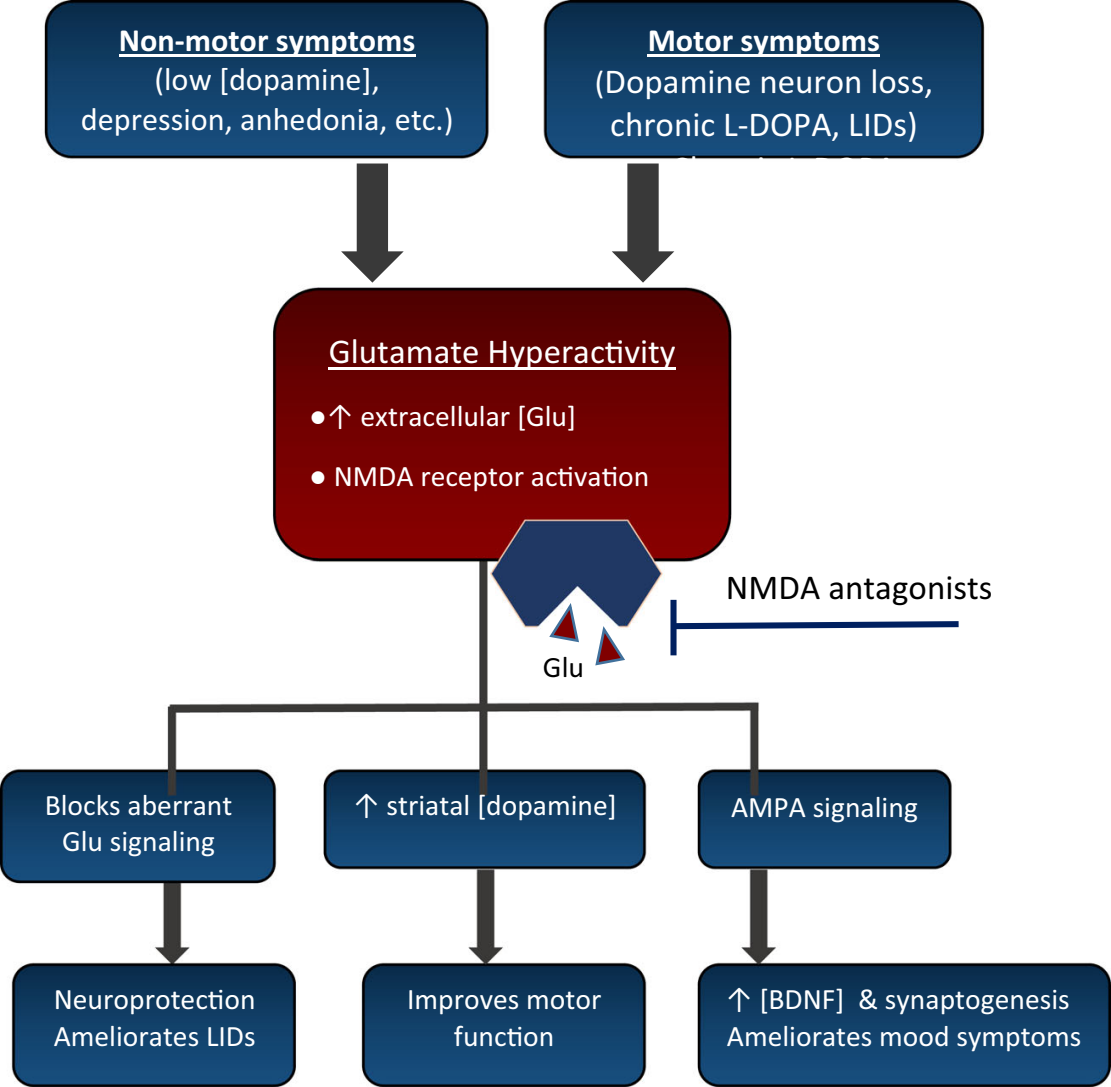
The motor and non-motor symptoms in Parkinson’s disease are hypothesized to arise from similar mechanism(s) involving a loss of dopamine input, leading to Glu hyperactivity. NMDA antagonists block hyperactive Glu binding with NMDA receptors and exert ameliorating effects on mood and motor function. Glu glutamate, LIDs levadopa-induced dyskinesias, AMPA 2-amino-3-(5-methyl-3-oxo-1,2-oxazol-4-yl)propanoic acid, BDNF brain-derived neurotrophic factor

The use of NMDA antagonists in PD is supported by three observations: (1) blockade of aberrant glutamate signaling in the subthalamic nucleus is crucial in the pathogenesis and motor PD symptoms, (2) subthreshold doses of NMDA antagonists synergize with Parkinsonian and dopaminergic agents^21^ by causing enhanced release and turnover of striatal dopamine^21^, and (3) PD models suggest that NMDA antagonism may protect nigral neurons^21,22^ (Fig. 2). It has been demonstrated that not only does NMDA antagonism improve PD symptoms, but may also be neuroprotective, preventing disease progression by inhibition of glutamatergic-mediated excitotoxicity^23^, and stimulating synaptogenesis/neurotrophic release^24,25^.

### Drugs for neuropsychiatric PD symptoms

Co-morbid depressive symptoms in PD patients are detrimental to daily life activities and there are indications that depression exacerbates cognitive and motor impairments in PD^26^. The impact of depressive symptoms, and the relationship between mood circuits, cognitive circuits, and PD, makes a compelling case for initiating treatment. Unfortunately, the response rate to first-line antidepressant medications is low among the geriatric PD population when compared to placebo^27^. Although selective serotonin reuptake inhibitors (SSRIs) are considered first-line therapy, their clinical efficacy in PD-dep is inconclusive^28^. In addition, SSRIs may lead to a worsening of motor symptoms, due to antagonistic effects on dopamine^29^. The Movement Disorder Society Task Force concluded that there was a lack of efficacious therapies in treating anxiety, apathy, and impulse control symptoms in PD^30^. There is a critical need for alternative pharmacological methods to treat PD-dep, as mainstream antidepressant drugs are largely ineffective in this population^27,31,32^. The use of memantine for treating cognitive dysfunction and other psychiatric symptoms (anxiety and depression) in PD was suggested by two small clinical trials^6,33^. There are anecdotal reports of PD subjects claiming “better mood” or “improved sense of humor” after memantine treatment^34^. Given the dual antidepressant and anti-dyskinetic effects of ketamine and other NMDA antagonists for PD, a number of NMDA antagonists will be reviewed for treating depression and other neuropsychiatric symptoms, including memantine, amantadine, ketamine, dizocilpine, and d-cycloserine. Ketamine is an agent that has been re-purposed for treating treatment-resistant depression^35^. The promise of ketamine in treating PD-dep is of particular interest. A recent case report series demonstrated the reduction of LIDs in PD patients after intravenous (IV) ketamine administration and the significant reduction in pain and depressive symptoms^36^. Although NMDA antagonists are classically utilized to reduce LIDs and motor symptoms in PD, the objective is to review the modulatory effects on neuropsychiatric symptoms. Given the few number of studies focused on PD-dep, behavioral and all cognitive symptoms will be included.

## Methods

A literature search was conducted using PubMed database. We used the following keywords: NMDA antagonist and Parkinson’s disease. We also searched the following databases up to March 1, 2018: Ovid MEDLINE, PsycINFO, CINAHL, Google Scholar, Cochrane Central Register of Controlled Trials, and Cochrane Database of Systematic Reviews. The initial search yielded 263 articles. The title and abstracts of the articles were then scanned for keywords, such as mood, psychiatric condition, depression, and cognition, yielding 40 full-text articles. The following criteria were used for inclusion: (1) articles in English or with an available published English translation, (2) publication in a peer-reviewed journal, (3) studies which quantitatively or qualitatively described the change in mood, QOL, sleep, cognition, or neuropsychiatric symptoms after NMDA antagonist therapy. Two authors (B.V. and W.O.) independently reviewed the articles identified from the search using the above selection criteria. After a review of full-text articles, 20 articles were excluded since they were not in English (*n* = 4), did not describe the change in neuropsychiatric symptoms before and after therapy (*n* = 10), or were literature reviews (*n* = 6). The two authors reached consensus on the 20 studies included.

## Results

### Description of included studies

Of the 20 articles included in this review, there were eight randomized placebo-controlled trials, one double-blind placebo-controlled study, four open label studies, one washout placebo-controlled trial, two case reports, and three animal studies. The pre-clinical studies included rat and monkey PD animal models. The neuropsychiatric symptoms that were described include depressive symptoms, irritability, anxiety, sleep, QOL, global neuropsychiatric inventory scores, executive function, and changes in cognition. The findings of the studies are listed in Table 1. For the full descriptions of the acronyms of the various clinical scales used, refer to Table 2 and for descriptions of each drug, refer to Table 3.

**Table 1:** List of articles on NMDA antagonists and Parkinson’s disease in PubMed Database (1969–2018)

Study (year)	Drug, dose, duration	Population characteristics	Design	Measures	Findings	Limitations	Bias score
Aarsland et al. (2009)	Memantine 20 mg/day 24 weeks	PD or LBD *N* = 72	Randomized, double-blinded placebo-controlled	CGI, MMSE, NPI, UPDRS	CGI score improved in patients given memantine (*p* = 0.03). Improved speed and global cognition led to the changes in CGI score. MMSE improvement (*p* = 0.02) although no significant differences in UPDRS and NPI scores.	High patient attrition and lacked adequate statistical power-grouped PD and DLB cohort	2
Wesnes et al. (2014)	Memantine 20 mg/day 24 weeks	Disrupted episodic memory/cognition *N* = 30 PDD	Randomized, double-blind, placebo-controlled multi-center	CDR	Memantine produced statistically significant medium to large effect-sized improvements in attentional performance involving information processing and to verbal episodic recognition memory as evidenced by increased performance in choice reaction time, immediate, and delayed word recognition.		2
Emre et al. (2010)	Memantine 20 mg/day 24 weeks	PD *N* = 120	Multi-center, randomized, double-blind, placebo-controlled	ADAS, NPI	NPI did not improve in PD (−1.6 vs. −0.1, respectively, difference of −1.4, *p* = 0.522) nor with ADAS (*p* = 0.576).	Small sample size, missing data at some time points	2
Leroi et al. (2009)	Memantine 20 mg/day, 16 weeks Washout and follow-up after 6 weeks (22-week)	PDD *N* = 25	Randomized, double-blind, placebo-controlled	NPI, DRS, MMSE	NPI and MMSE changes between memantine and placebo were insignificant (sub-scores not shown). DRS changes between placebo (100.3) in favor of memantine (94.7). After washout, memantine group more often had global deterioration (70%) vs. (29%) for placebo.	NPI sub-scores not shown, small sample size, some participants on cholinesterase inhibitors before trial	2
Litvinenko et al. (2008)	Memantine 20 mg/day 12, 24 week assessment	PD *N* = 62	Placebo-controlled	ADAS-cog, Verbal Fluency Test, clock-drawing test NPI, FAB, DAD	At 24 weeks, memantine group had significant improvements in ADAS-cog (0.002), FAB (*p* = 0.01), verbal fluency test (*p* = 0.01), and clock drawing test (*p* = 0.03). NPI improvement with memantine, i.e., disinhibition (*p* = 0.006), irritability (*p* = 0.004), anxiety (*p* = 0.04), and hallucinations (*p* = 0.048). No significant changes at 12 weeks. At 52 weeks, significant improvements in all scales.	Non-randomized, non-blinded	2
Johansson et al. (2010)	Memantine 20 mg/day 24 weeks 4 week washout additional 26 week trial	PD and LBD *n* = 56	Washout and open label continuation after a double-blind randomized controlled trial	CGIC, presence of recurrence of symptoms upon drug withdrawal	Washout of memantine caused 9% (attrition due to worsening of psychiatric symptoms, i.e., anxiety and depression). CGIC scores worsened more in the memantine washout group vs. placebo.	Grouped PD and DLB cohort	2
Vidal et al. (2013)	Memantine 20 mg/day ~ 3 months	PD *N* = 2	Case report	Global UPDRS (I–III)	Global UPDRS showed improvement following treatment with memantine with the unanticipated improvement in LID and on–off phenomenon. For patient #2 = 4 point improvement in mood/behavior, no change in patient #1.	Small sample size	2
Leroi et al. (2014)	Memantine 20 mg/day 22 weeks	PDD *N* = 25	Randomized, double-blind, placebo-controlled	GAS, PDQ-8, Zarit Burden Inventory	A significantly greater proportion of participants on memantine (64%) had better than expected GAS outcomes compared with those on placebo (7%) (*p* = 0.007). GAS score, as well as mean caregiver burden score, from baseline to drug discontinuation was significantly greater in memantine compared to placebo (*p* = 0.03 and 0.04, respectively). Although significant differences in QOL were not seen, memantine improved individually set goals and caregiver burden in PDD.	Ungeneralizable outcome measures, small sample size, no measure of inter-rater reliability for GAS	2
Larsson et al. (2011)	Memantine 20 mg/day 24 weeks	PDD or LBD *N* = 70	Randomized, double-blind, placebo-controlled	Caregiver-rated QOL-Alzheimer’s Disease in domains (WHO health classification)	Memantine significantly improved total QOL in both PDD and LBD patients (*p* = 0.01), memantine had 42% rate higher QOL than at baseline compared to 15% in placebo.	Small sample size, some patient attrition, possible response shift phenomenon	2
Ondo et al. (2011)	Memantine 20 mg/day 8 weeks	PD *N* = 40	Double-blind placebo-controlled exploratory pilot trial	UPDRS I–II ESS, HAM-D, QOL-39, CGI	No significant change in UPDRS section I or II, Epworth sleepiness scale, fatigue severity scale, Hamilton depression scale, Conner adult inventory, PD Quality of Life-39, or clinical global impressions.	Short time period, only 8-week follow-up	1
Larsson et al. (2010)	Memantine 20 mg/day 24 weeks	PDD and LBD Excessive daytime sleepiness and REM disorder in PDD *N* = 20 (PDD + DLB)	Randomized, double-blind, placebo-controlled	Stavanger sleep questionnaire and ESS	At 24 weeks, patients treated with memantine were less physically active during sleep while patients in the placebo group worsened (*p* = 0.006). No significant change was observed in severity of excessive daytime sleepiness.	Small sample size, some patient attrition, possible awareness bias due to methodology	2
Pahwa et al. (2015)	Amantadine ADS‐5102 extended-release (260–420 mg/day) 8 weeks	PD patients with levodopa-induced dyskinesia *N* = 83	Randomized, double-blind, placebo-controlled, multisite study	UPDRS (I–III), PDQ-39	Insignificant change in UPDRS and QOL/PDQ-9 at week 8 for any dose.	UPDRS I score combined with II–III so effect on mood cannot be determined Focus was on safety of novel formulation (extended release)	1
Bandini et al. (2002)	Amantadine 100 mg/day/1st week 200 mg/day/2nd week 300 mg/day/3rd week 6 months	PD *N* = 23 Cognition and visual response	Open-label cohort study with amantadine monotherapy vs. adjuvant levodopa	Event related P300 (visual discrimination paradigm)	Amantadine alone and as adjuvant to levodopa can significantly improve both the speed of visuo-cognitive processing and shortened latency of visual discrimination (*p* < 0.05).	Small sample size, non-randomized, single ethnicity (African-American)	2
Parkes et al. (1970)	Amantadine 100 mg/day (2 weeks) 300 mg/day (2 weeks) 500 mg/day (2 weeks) 6 week trial	PD *N* = 43	Cohort study	Self-designed motor, sensory and psychological tests, patient report	During trial, 26/43 patients reported improved “mood”, regardless of dose.	Non-randomized, non-placebo-controlled, small sample size, unverified techniques to measure PD symptoms	0
Schwab et al. (1969)	Amantadine 100–200 mg/day 6 months	PD *N* = 163	Cohort study without a placebo control	Patient report	20% of patients experienced side effects of increased jitteriness, insomnia, abdominal uneasiness, loss of appetite, and slight subjective dizziness. One patient reported a feeling of depression. 22% of patients experienced insomnia, dizziness, confusion. One patient reported depression after amantadine.	Non-randomized, non-placebo-controlled, no description of how symptoms were quantified following drug administration	1
Sherman et al. (2016)	Ketamine 0.15–0.3 mg/kg/h 50–96 h periods	PD *N* = 5 Pain and depression	Case reports	Patient report	Depression and suicidality improved in one depression-positive patient (measure is not stated). However, the patient continued to exhibit mild symptoms of depression in follow-up visits. Pain improved for all five patients: two back pain, two headache and one painful dyskinesia.	Small sample size, multiple unspecified metrics used to measure outcomes	1
Schneider et al. (2000)	D-cycloserine 320, 1000, 8000 µg/kg Dizocilpine (10–32 µg/kg) (single)	MPTP-induced PD primate model *N* = 4	Animals served as their own controls with and without treatment	Performance of a variable-delayed-response task	D-cycloserine significantly improved performance of a variable-delayed-response task. No effect at cycloserine 8000 µg/kg or with dizocilpine.	Small sample size, animal model	^a^
Ho et al. (2011)	D-cycloserine 30 mg/kg/day 100 mg/kg/day 200 mg/kg/day (13 days)	MPTP-induced PD rat model	Animals served as their own controls	Rotarod test, T-maze, plus-maze	Treatment with D-cycloserine improved deficits in working memory and anxiety-like behavior.	Small sample size, animal model	^a^
Singh et al. (2017)	Dizocilpine 0.2 mg/kg, ip	6-OHDA-induced PD rat model anxiety and depression	Experimental	Light chamber (time spent) social interaction test (anxiety-like behavior) immobility test (depression)	Dizocilpine-treated rats spent more time in the light chamber (*p* < 0.01), and more contact time during the social interaction test (*p* < 0.05) in comparison to 6-OHDA lesioned rats.	Animal model	^a^
Montastruc et al. (1994)	Dextromethorphan 90–180 mg/day 1 month	PD *n* = 10 global symptoms	Open label	UPDRS (I–III)	No significant improvement in UPDRS subscores of extrapyramidal symptoms (partial tremor, rigidity, bradykinesia) mentation, behavior, and mood or daily activity.	Small sample size, specific changes in UPDRS score not provided	^a^

Quality assessment criteria for selected studies		
Score	Method for measuring non-motor symptoms	Trial period
1	Subjective, self-reports by patient or qualitative noting by clinician	Adequate time period
0	Objective measure, either by clinician (UPDRS-1, MMSE, ADAS, etc.) or by caregiver (NPI)	Inadequate time period, less time than average trials for given drug

Abbreviations: *ADAS-Cog* Alzheimer’s disease assessment scale-cognitive, *CDR* clinical dementia rating, *CGI* clinical global impressions, *DAD* disability assessment for dementia, *ESS* Epworth sleepiness scale, *FAB* frontal assessment battery, *GAS* goal attainment scaling, *HAM-D* Hamilton depression rating scale, *MMSE* mini-mental state examination, *PDQ-8/39* Parkinson’s disease questionnaire-8/39-item, *NPI* neuropsychiatric inventory, and *UPDRS* unified Parkinson’s disease rating scale

^a^ Bias assessment was not completed with animal studies

**Table 2:** Description of the scales and measures commonly used in studies

Scale	Description
Alzheimer’s disease assessment scale-cognitive (**ADAS-cog**)	The ADAS-cog is a frequently used test that measures cognition in clinical trials for new medications, interventions, and in research studies. It is comprised of 11 parts that primarily measures memory and language. It was developed as an outcome measure to antidementia therapies as a two-part scale: one that measures non-cognitive functions and another that measures cognitive functions.
Clinical dementia rating **(CDR)**	The CDR is a 5-point scale that assesses cognitive and functional performance of individuals with Alzheimer disease and related dementias. It characterizes six domains—memory, personal care, community affairs, home and hobbies, orientation, and judgment and problem solving. The information to rate each is collected via an interview and reliable collateral sources.
Clinical global impressions **(CGI)**	Overall clinician-determined summary that measures symptom severity and treatment response. It also provides a brief summary of the clinician’s assessment of a patient with a mental disorder before and after starting a study medication. The summary considers the patient’s history, symptoms, behavior, psychosocial circumstances, and how the symptoms impact a patient’s functionality.
Disability assessment for dementia **(DAD)**	The DAD scale is an informant-based interview that includes instrumental and basic ADL items used to diagnose and assess patients with dementia or MCI. It evaluates the activities that are problematic followed by addressing aspects of performance that are impaired.
Dementia rating scale (**DRS)**	The dementia rating scale is designed to evaluate the level of cognitive functioning for persons with brain dysfunction. It is a 36-task and 32-stimulus card individually administered instrument capable of differentiating the extent of deficit and is also sensitive at the lower ends of functioning.
Epworth sleepiness scale **(ESS)**	The Epworth sleepiness scale is a questionnaire that measures an individual’s overall level of daytime sleepiness.
Frontal assessment battery **(FAB)**	The FAB consists of six neuropsychological tasks designed to explore the behavioral and cognitive domains of executive functioning and thereby assess frontal lobe function at bedside. The six domains tested are inhibitory control, environmental autonomy, conceptualization, mental flexibility, self-regulation and resistance to interference, and motor programming and executive control of action.
Goal attainment scaling (**GAS**)	Idiographic approach for measuring outcomes of psychosocial interventions in community settings. The patient’s goals are assigned to a behavioral expectation that ranges from a point scale of best (+2) to worst possible outcome (−2)^1^.
Hamilton depression rating scale **(HAM-D)**	The HAM-D is a multiple item questionnaire designed for adults to rate the severity of their depression and is also used as a means to assess recovery. It is the most commonly used scale to evaluate the efficacy of antidepressant therapy via symptom severity.
Mini-mental state examination (**MMSE)**	This is a 30-point questionnaire used in research and clinical settings in order to measure cognitive impairment, estimate progression and severity of cognitive impairment, and follow the course of cognitive changes over time for each individual. It is an effective method to record an individual’s response to treatment, by examining orientation, registration, attention and calculation, recall, language, and ability to follow simple commands.
Parkinson’s disease questionnaire-8 **(PDQ-8)**	The PD questionnaire-8 is a self-administered shortened version of Parkinson disease questionnaire-39 that consists of one selected item from each of the 8 quality of life dimensions of the PDQ-39. It is less time consuming, more easily administered, and measures the quality of life for individuals with Parkinson’s disease.
Parkinson’s disease quality of life scale-39 **(PDQ-39)**	The PDQ-39 is a 39-item self-report comprehensive Parkinson’s disease assessment questionnaire that evaluates how patients experience difficulties, and the impact of Parkinson’s disease (PD) across eight specific dimensions of functioning and well-being. It is based on statistical criteria of 39-multiple-choice items covering 8 dimensions, mainly used in clinical trials.
Neuropsychiatric inventory **(NPI)**	The NPI is a structured, caregiver-based interview designed to detect, quantify and assess changes of psychiatric symptoms within a demented population. It evaluates 10 behavioral domains—delusions, hallucinations, agitation/aggression, anxiety, irritability, euphoria, apathy, dysphoria/depression, disinhibition, and aberrant motor behavior. Often, two other domains are included—weight changes and nighttime behavioral disturbance (NPI-12). A lower score is better.
The unified Parkinson’s disease rating scale **(UPDRS-I & II)**	The UPDRS follows the longitudinal course of Parkinson’s disease and it is the most commonly applied rating scale for PD. Higher score signifies more severe Parkinsonism. Clinicians utilize it to follow the progression of individuals with PD, while researchers use it to measure changes from interventions. It is comprised of 31 items contributing to three subscales: (I) Behavior, Mentation, and Mood; (II) Activities of Daily Living; and (III) Motor Examination. It provides insight to the patient’s disease progression while sensitive to change over time.
Verbal fluency test	The verbal fluency test is a psychological test whereby patients categorically produce as many words as possible within a certain time frame. The category can be phonemic such as words beginning with a specific letter, or semantic like an object. Even though the total number of words is used to measure performance, other analyses like length and number of clusters of words from the same subcategory or number of repetitions can be performed.
Zarit burden inventory **(ZBI)**	The ZBI is a measure of caregiver burden for individuals with dementia. Multiple versions have been published, which feature statements that are ranked by informants, with higher scores reflecting greater caregiver burden. It has also been used in a number of other applications, such as outcome measures for drug trials and specific patient groups.

**Table 3:** Description of NMDA antagonists and mechanism of action(s)

Drug, indication	Mechanism of action
Dextromethorphan, rapid antidepressant	Putative NMDA-2A/2B-receptor antagonist NMDA-3A antagonist Opioid sigma 1 and sigma 2 receptor agonists mTOR activation Alpha 3/beta 4 nicotinic receptor antagonist Targets the serotonin reuptake pump Putative AMPA activation
Ketamine, rapid antidepressant and pain, anesthetic agent	NMDA-3A receptor antagonist Substance *P* receptor antagonist—probably associated with G proteins that activate a phosphatidylinositol–calcium second messenger system D2 dopamine receptor agonist/partial agonist Kappa-opioid receptor agonist; Mu/delta-opioid receptor binder 5-hydroxytryptamine receptor 1 & 2 antagonist Muscarinic acetylcholine receptor binder Induction of BDNF expression mTOR modulation and activation
Amantadine, treats drug-induced extrapyramidal reactions	NMDA receptor antagonist D2 receptor agonist—mediated by G proteins which inhibit adenylyl cyclase Matrix protein 2 inhibitor for Influenza A virus
Memantine, treats moderate to severe cognitive impairment	NMDA-3A antagonist NMDA-2A/2B-receptor antagonist NMDA-1 binder D2 dopamine receptor agonist 5-hydroxytryptamine receptor 3A antagonist Alpha-7 nicotinic cholinergic receptor subunit antagonist
D-cycloserine, second-line agent for drug-resistant tuberculosis; cognition enhancer	Putative cyclic NMDA partial agonist Glycine site partial agonist Inhibits cell-wall biosynthesis in bacteria Alanine racemase inhibitor
Dizocilpine (MK-801), potent anticonvulsant	Noncompetitive NMDA receptor antagonist Inhibits reuptake of dopamine, noradrenaline, and serotonin Nicotinic acetylcholine receptor antagonist

### Memantine

There were 11 human studies that described the neuropsychiatric symptoms with memantine treatment. All trials were conducted at 20 mg/day, unless noted otherwise.

The effect of memantine on cognition was assessed in five randomized controlled trials (RCTs), and measured by the mini-mental state examination (MMSE), neuropsychiatric inventory (NPI), or clinical dementia rating (CDR) scales. In a 24-week trial, the memantine group had a slight, yet significant improvement in cognition on the MMSE (*p* = 0.02). Overall, there was a greater improvement in the memantine group as 27% of patients experienced a moderate to substantial improvement and no placebo patients reported more than a slight improvement. However, there was an insignificant change in NPI scores^37^. In a separate 24-week trial, 30 PD patients experienced improvements in cognition with medium to large effect sizes in information processing and recognition memory^38^. In a 24-week trial with memantine, the NPI scores did not significantly differ in PD patients (*p* = 0.522)^39^. Although there were notable improvements in items such as apathy, anxiety, irritability, and depression, the improvements did not reach statistical significance when compared to placebo. Cognitive scores were insignificant for PD (*p* = 0.576). A placebo-controlled 24-week trial of memantine improved cognition among 32 PD patients, as evidenced by various scales, including the Alzheimer’s disease assessment scale-cognitive (0.002), the frontal assessment battery (*p* = 0.01), verbal fluency test (*p* = 0.01), and clock drawing test (*p* = 0.03). Notably, the improvement in NPI sub-sections included disinhibition (*p* = 0.006), irritability (*p* = 0.004), anxiety (*p* = 0.04), and hallucinations (*p* = 0.048) when compared to placebo. The most significant change was caused by the decreased disinhibition/impulsive behavior in four patients. The number of patients who improved was not specified among the other NPI sub-sections. There was no significant change or improvement with memantine at 12 weeks^6^. When treatment was extended to 52 weeks, there were significant improvements and enhancement on cognition and neuropsychiatric symptoms on all scales (*p* < 0.05).

The cognitive effects of memantine discontinuation were shown in a 22-week trial by Leroi et al., 25 PD patients were entered in a washout phase after a 16-week trial with memantine. After the washout phase, patients were re-assessed at a 6-week follow-up. At follow-up, the improvement in cognition was only evident with the dementia rating scale (DRS), with insignificant differences between memantine and placebo with the NPI and MMSE scales, the sub-scores for each scale were not shown. At follow-up, the memantine group also had more global deterioration (70%) than placebo (29%)^33^. In a washout trial and post 24-week treatment with memantine, the attrition rate was most affected by the worsening of anxiety and depressive symptoms (9%). The clinical global impressions (CGI) scores also considerably worsened in the memantine washout group vs. placebo^40^. A case report (*n* = 2) described an improvement on mood/behavior for one patient after 3-month treatment, but no change in the other patient, measured by the unified Parkinson’s disease rating scale (UPDRS)-I section^41^.

The changes in QOL in PD patients after memantine treatment were insignificant in an 8-week trial^34^ and in a 22-week trial^42^, but significant improvements were observed in a 24-week trial in which the memantine group had a 42% higher QOL compared to 15% placebo (*p* = 0.01)^43^. In terms of sleep quality, an 8-week exploratory pilot trial by Ondo et al. did not show a significant change in sleep^34^, while the 24-week trial observed improved sleep and less physical activity during sleep compared to placebo (*p* = 0.006), without affecting daytime sleepiness^44^. The Ondo et al. trial recorded insignificant changes in other mood metrics, such as in UPDRS-I, II, the fatigue severity scale, and the Hamilton depression scale^34^.

### Amantadine

There were four studies that described the neuropsychiatric symptoms after amantadine treatment. The safety and efficacy of extended release amantadine (260, 320, and 420 mg/day) were established in an 8-week randomized, double-blind controlled trial. Although the formulation was well-tolerated, there were insignificant changes in the global UPDRS and QOL scores at any dose. However, the mood score on the UPDRS-I section was unknown since it was reported as one global score, and incorporated the II–III sections^45^. The following three studies were open-label, non-randomized, and not placebo-controlled. Only one study had a cognitive measure, in which the visual and cognitive processing significantly improved after a 6-month trial of tapered amantadine (100–300 mg/day)^46^. During the course of a 6-week trial of tapered amantadine (100–500 mg/day), 26 of the 43 PD patients subjectively reported an “improved mood”, regardless of amantadine dose. There were no objective measures in the study^47^. Lastly, psychiatric adverse events were reported in a 6-month trial of amantadine (100–200 mg/day), as 20% of patients experienced increased jitteriness, insomnia, abdominal uneasiness, loss of appetite, and one patient developed depression^48^. These adverse symptoms promptly disappeared within 36 h of drug cessation. The authors commented that amantadine potentiated side effects of belladonna-like drugs, as those patients who reported adverse events were concurrently taking trihexyphenidyl (Artane) and benztropine (Cogentin)^48^.

### Ketamine

Ketamine is an agent that has been re-purposed for treating mood disorders and effective at treating major depression^49^. Though there are a few studies discussing the use of ketamine for management of PD dyskinesias^50,51^. There was only one study to describe behavioral symptoms by researchers at Arizona Tucson University^36^. Five PD patients were treated with ketamine for a constellation of intractable pain and painful dyskinesia, and one patient had suicidal ideation and depression. Among both patients whose pain was assessed, there was a 50% decrease in pain after ketamine infusion (pain scale assessment, 1–10). The dose range was 0.05–0.15 mg/kg for 65 h or 96 h. Severe depressive symptoms and suicidality in one patient improved to “mild” depression after a 65 h continuous ketamine infusion at an average dose of 0.09 mg/kg/h. No metrics or scales were used to determine improvements in depression. This study suggests that low-dose sub-anesthetic ketamine infusions are well-tolerated and safe in a PD population.

### D-cycloserine

There were two animal studies that measured the effect of d-cycloserine on behavioral and cognitive performance. In both studies, animals were challenged with chronic administration of 1-methyl-4-phenyl-1,2,3,6-tetrahydropyridin (MPTP) to induce a PD model and subsequent administration of d-cycloserine to assess the extent of recovery in PD symptoms. The cognitive-enhancing ability of d-cycloserine for PD was suggested in a primate model after Schneider et al. treated primates with a single administration of d-cycloserine (320 or 1000 µg/kg), and there was a significant improvement in a variable-delayed-response task. However, there was no effect at a high dose of d-cycloserine (8000 µg/kg)^52^. In a PD rat model, Ho et al. observed improved cognition and memory after intraperitoneal injection of d-cycloserine across 13 days. In addition to memory, anxiety-like behavior decreased, and was observed by a number of behavioral animal tests, including the rotarod test, T-maze, and plus-maze^53^.

### Dizocilpine

In a primate PD model, a single administration of dizocilpine (10–32 µg/kg) had no effect on cognitive improvement^52^. A PD rat model was challenged with the neurotoxin 6-hydroxydopamine (6-OHDA), and produced anxiety and depressive-like behavior. After intraperitoneal dizocilpine (0.2 mg/kg), rats spent more time in the light chamber (*p* < 0.01), and more contact time during the social interaction test (*p* < 0.05) in comparison to 6-OHDA lesioned rats. This suggests that MK-801 ameliorates depressive-like behavior in 6-OHDA lesioned rats^54^.

### Dextromethorphan

In an open-label pilot study of 10 PD patients, a daily dose of 90–180 mg/day of dextromethorphan did not significantly improve UPDRS sub-scores in mentation, behavior, and mood domains. The follow-up was at 1 month^55^.

## Discussion

### Cognitive dysfunction

Neurocognitive dysfunction and dementia can occur in up to 40% of PD patients, with dementia onset being more associated with disease progression^56^. Similar to Alzheimer’s dementia, it is suggested that impaired cholinergic pathways are the cause of PD dementia^57^, however, this may be related to aberrant glutamate activity^58^. In addition to improvements in motor symptoms, there is preliminary evidence that NMDA antagonists may be effective at treating PD psychiatric symptoms. In most studies, NMDA antagonists significantly improved cognition as the primary outcome (*n* = 4)^6,37,38,46^. Drug discontinuation and washout with memantine caused significant global deterioration in cognition and overall neuropsychiatric symptoms, which may be indicative of the effectiveness during the dosing period (*n* = 2)^33,40^. The exceptions were two memantine studies that showed moderate improvement in cognition, but were not statistically significant when compared to placebo^33,39^. The utility of memantine may be specific for cognition and plays a minimal role for other psychiatric symptoms. For example, a single study showed significant improvements with the MMSE measure, but not with NPI or UPDRS, measuring changes in neuropsychiatric and global PD symptoms, respectively^37^. A systematic review and analysis compared memantine to cholinesterase inhibitors in treating PD dementia and found that both drugs significantly improved global impression; however, cholinesterase inhibitors were more effective at enhancing cognitive function than memantine^59^. Although less commonly used in PD dementia, amantadine showed promise in enhancing cognition, especially in improving the visual-cognitive processing and visual discrimination^46^. The therapeutic effect of longitudinal use of amantadine was suggested in an 8-year study, revealing that amantadine delayed the onset of PD dementia by ~ 3.2 years and attenuated dementia severity in a dose-response manner^60^. While other NMDA antagonists, such as d-cycloserine improved cognitive scores, the disadvantage is it has only been shown in PD animal models^52,53^. In summary, there is potential for NMDA antagonists not only in treating LIDs, but also in attenuating dementia.

### Depressive and anxiety symptoms

For many of the included studies, changes or improvements in mood were qualitative. However, when measures were used, the NPI or the UPDRS-I was utilized to measure depression, anxiety symptoms, and “general behavior mentation and mood”. The NPI contains a wide inventory of psychiatric sub-score symptoms, such as anxiety, depression, and irritability. The use of amantadine for treating neuropsychiatric symptoms was first suggested in 1968, when it was noted that the drug appeared to produce a positive effect and a feeling of general well-being and affect among PD patients^48^ and 2 years later, self-reports of “improved mood” emerged among a PD cohort^47^. Memantine appeared to have improved anxiety and irritability symptoms to a greater extent, than other psychiatric symptoms described in the NPI^6^. One study described the anecdotal reports of PD patients endorsing a “better mood” with memantine^41^. Three memantine studies offset these observations by not showing a significant difference in global NPI scores between treatment and placebo^37,39^. The disadvantage was that only the global NPI scores were reported; there could have been significant changes in sub-scores, yet not in the global score. It is of clinical interest to report changes in these domains that are indicative for specific symptoms (anxiety and depression) between studies. Future studies would be strengthened by reporting of NPI sub-scores/domains. However, measuring depressive symptoms utilizing a semi-structured interview, such as the HAM-D scale, did not show an improvement. However, this trial was only 8-weeks long with memantine^34^, as opposed to the more common period of 24 weeks. The beneficial effect on mood may take longer, as a similar study showed that NPI scores improved at 24 weeks, but not at 12 weeks^6^. Memantine has shown previous success at treating obsessive-compulsive syndromes^61,62^. Notably, memantine has markedly decreased impulsive behavior in PD patients^6,63^, which may be explained in terms of the glutamatergic dysfunctions in the lateral orbitofrontal circuit. This same study showed a significant improvement in anxiety at 52 weeks, but not at 12 weeks. Washout of memantine can cause worsening of anxiety and depressive symptoms^40^. These detrimental effects observed during washout may also be indirectly indicative of memantine’s effectiveness.

The efficacy of ketamine as an antidepressant and its application for major depressive disorder is under active investigation. To date, there have been nine high-quality RCTs, which have documented the markedly high response rate in the ketamine intervention group, when compared to placebo^49^. Notwithstanding some limitations, ketamine is a promising therapy for treatment-resistant depression^35,64^ and may resolve suicidal thoughts in patients experiencing suicidal ideation after a single infusion^65^.

A continuous ketamine infusion resolved suicidal thoughts and severe depression in a PD patient. The patient’s dyskinesia and pain symptoms were also resolved after ketamine^36^. This highlights the efficacy of a single drug to treat a constellation of PD symptoms and the ability to improve QOL. Other researchers have started similar initiatives^66^. Given the aberrant NMDA signaling in PD pathology, the use of NMDA antagonists such as ketamine may be a viable therapeutic option. Although the initial results are promising, large-scale clinical trials will be needed to determine the efficacy and safety in PD^64^. The efficacy of ketamine has spurred the development of alternate glutamate modulating antidepressants, such as rapastinel^67^. An advantage of newer therapeutics such as rapastinel is the induction of antidepressant effects without the negative psychoactive side effects of ketamine^68^. To note, although there are antidepressant effects of other NMDA antagonists such as dextromethorphan^69^, there is no literature of their effectiveness in PD-dep. There are reports of neuroprotection after acute dextromethorphan in a number of PD models^52,70^. A recent study showed improvement in anxiety and depressive symptoms in a PD rat model with dizocilpine treatment, which was speculated to have occurred via Wnt signaling^54^. In summary, the use of NMDA antagonists is becoming more common in treating psychiatric symptoms, especially depression. It is likely that future therapeutics will become more focused on modulating the glutamate system.

### Overall quality of life

The non-motor PD symptoms are major predictors in the decline of QOL. There are few studies that measured QOL after NMDA antagonist therapy. One study showed a 42% higher QOL after 24-week memantine therapy than at baseline, compared to 15% of placebo^43^. Although promising, a significant QOL improvement was not attained in two other memantine studies^34,71^ and one amantadine study^45^. However, the amantadine study duration was only 8 weeks and it is suggested that improvements in QOL may not be apparent until longer follow-up. For example, the change in QOL was insignificant with memantine at 8-week follow-up^34,45^, but significantly improved at 24 weeks^43^. There is an evidence that memantine may not only improve QOL, but may also have a disease-modifying effect. A longitudinal study of 227 newly referred PD patients found that non-motor symptoms’ burden and QOL were predictive in PD progression over a 2-year period. Specifically, sleep/fatigue, mood/apathy, and attention/memory domains were most significantly predictive of QOL changes^72^. Similar studies were conducted in PD patients in Northeastern Mexico and Taiwan. Specifically, sleep/fatigue, mood/cognition, and gastrointestinal domains were associated with worse QOL in the Mexican PD population^73^. For Taiwanese PD patients, the depression/anxiety item was strongest, where disease duration and severity, but not pharmacological therapy, were major predictors of non-motor symptoms^74^. Further development and evaluation of interventions that improve QOL are needed. Long-term follow-up studies show that the survival time was significantly higher in PD patients who were on memantine trials (*p* = 0.045)^75^. It is suggested that an early positive response to memantine may be a prediction factor in longer survival. Therefore, the clinical use of memantine and amantadine for improving QOL may be beneficial in disease progression.

### Other considerations

The positive effects of memantine in PD symptoms may be explained by indirect effects of NMDA antagonism. It is suggested that NMDA antagonists may offer neuroprotection by counteracting the hyperactive metabolism in PD basal ganglia. In a cohort study, PET scans of PD patients on 6-week memantine treatment showed neuronal modulation in the basal ganglia with decreased regional cerebral blood flow in the basal ganglia^76^ and by blocking excitotoxicity^77^. The use of NMDA antagonists as mainstream medications for PD treatment is supported by various factors: (1) improving LIDs by pharmacological synergism with dopaminergic agents (i.e., L-DOPA), thereby, potentially decreasing the effective dose of L-DOPA^21,78^, (2) improving basal motor function by endogenous release of striatal dopamine in vivo^79,80^, and (3) offering possible neuroprotection in the context of overactive glutamatergic neurotransmission^21,22^ and possible alleviation of psychiatric symptoms, discussed in this review. However, widespread use is limited by intolerable side effects and the pharmacology of current NDMA antagonists. For example, amantadine affects other neurotransmitter systems that need to be carefully monitored in PD patients, such as norepinephrine, serotonin, gamma-aminobutyric acid and acetylcholine^81^, while memantine (Namenda) is well-tolerated and has few side effects^82^. Future research will need to focus on specific antagonists that act on aberrant NMDA antagonism.

## Conclusion

The future of antidepressants will extend beyond modulating the serotonergic and dopaminergic neurotransmitter systems. The repurposing of existing NMDA antagonists, such as ketamine, for depression and newer therapies, such as rapastinel, highlight the movement toward modulating the glutamatergic system. There is preliminary evidence that NMDA antagonists may modulate psychiatric symptoms in PD. However, the current evidence is inconclusive, and further trials must be conducted to elucidate their psychiatric-modifying effects.

### Strengths and limitations

Given the novel application of NMDA antagonists for treating non-motor PD symptoms, a broad approach was taken in reviewing the literature. This allowed for the discussion of preclinical models and self-reported or anecdotal symptom reports by patients and clinicians. One common limitation in the included trials was the use of concurrent medications. Many studies included patients concurrently taking L-DOPA and other dopaminergic-modulating PD drugs. This may confound the therapeutic effect of NMDA antagonists on mood. However, this may enhance the applicability to naturalistic settings. Another confounding factor was the heterogeneity of the study population. Especially relevant in the cognitive studies, patients with PD and Lewy body dementia (LBD) were treated and analyzed simultaneously. Notably, memantine appeared to fare better among LBD patients than in PD, as evidenced by greater improvements in CGI and NPI scores. However, there are reports of worsening of psychotic symptoms with memantine in advanced LBD, though it may be exacerbated due to multiple psychotropic medications^83^. These findings suggest that LBD and PD-dep are sufficiently different to warrant different dosing strategies with memantine. When possible, results from only the PD cohort were reported in this review.
